# Knockdown of EphB1 receptor decreases medulloblastoma cell growth and migration and increases cellular radiosensitization

**DOI:** 10.18632/oncotarget.3369

**Published:** 2015-03-30

**Authors:** Shilpa Bhatia, Nimrah A. Baig, Olga Timofeeva, Elena B. Pasquale, Kellen Hirsch, Tobey J. MacDonald, Anatoly Dritschilo, Yi Chien Lee, Mark Henkemeyer, Brian Rood, Mira Jung, Xiao-Jing Wang, Marcel Kool, Olga Rodriguez, Chris Albanese, Sana D. Karam

**Affiliations:** ^1^ Department of Radiation Oncology, University of Colorado Denver, Anschutz Medical Campus, Aurora, CO 80045, USA; ^2^ Department of Oncology, Lombardi Comprehensive Cancer Center, Georgetown University Medical Center, Washington, DC 20057, USA; ^3^ Sanford-Burnham Medical Research Institute, La Jolla, CA 92037, USA; ^4^ Department of Pediatrics, Emory University School of Medicine, Atlanta, GA 30322, USA; ^5^ Department of Developmental Biology, University of Texas Southwestern Medical Center, Dallas, TX 75390, USA; ^6^ Children's National Medical Center, Washington DC 20010, USA; ^7^ Georgetown University Hospital, Washington, DC, 20007, USA; ^8^ Department of Pathology, University of Colorado Denver, Anschutz Medical Campus, Aurora, CO 80045, USA; ^9^ Division of Pediatric Neurooncology, German Cancer Research Center DKFZ, Heidelberg, Germany; ^10^ Department of Pathology, Georgetown University School of Medicine, Washington, DC 20057, USA

**Keywords:** Eph, medulloblastoma, ATM, cell cycle, radiosensitization

## Abstract

The expression of members of the Eph family of receptor tyrosine kinases and their ephrin ligands is frequently dysregulated in medulloblastomas. We assessed the expression and functional role of EphB1 in medulloblastoma cell lines and engineered mouse models. mRNA and protein expression profiling showed expression of EphB1 receptor in the human medulloblastoma cell lines DAOY and UW228. EphB1 downregulation reduced cell growth and viability, decreased the expression of important cell cycle regulators, and increased the percentage of cells in G1 phase of the cell cycle. It also modulated the expression of proliferation, and cell survival markers. In addition, EphB1 knockdown in DAOY cells resulted in significant decrease in migration, which correlated with decreased β1-integrin expression and levels of phosphorylated Src. Furthermore, EphB1 knockdown enhanced cellular radiosensitization of medulloblastoma cells in culture and in a genetically engineered mouse medulloblastoma model. Using genetically engineered mouse models, we established that genetic loss of EphB1 resulted in a significant delay in tumor recurrence following irradiation compared to EphB1-expressing control tumors. Taken together, our findings establish that EphB1 plays a key role in medulloblastoma cell growth, viability, migration, and radiation sensitivity, making EphB1 a promising therapeutic target.

## INTRODUCTION

The erythropoietin-producing hepatocellular carcinoma (Eph) receptors constitute the largest family of receptor tyrosine kinases and are comprised of 14 different receptors and their cognate ligands, the ephrins [1, 3]. Eph receptors are divided into two classes: the EphA receptors, which interact preferentially with glycosylphosphatidylinositol (GPI)-linked ephrin A ligands; and the EphB receptors, which bind transmembrane ephrin B ligands. The transmembrane anchorage of ephrin B ligands permits both forward signaling, through the Eph receptor-expressing cell, and reverse signaling, through the ephrin-expressing cell. During mammalian development, members of the Eph/ephrin families are known to fulfill important roles in tissue formation and organization including axon guidance, synaptogenesis and pattern formation [1]. Given their importance in normal development, it is not surprising that the deregulation of normal Eph/ephrin signaling is involved in tumorigenesis [2, 3], with recent reports focusing on the effects of Eph/ephrin system on cell adhesion, migration, proliferation, and angiogenesis [4, 5].

Medulloblastoma is a primitive neuroectodermal tumor arising from granule neuron precursors in the cerebellum or from neural stem cells of the rhombic lip. These highly aggressive tumors are among the most frequently diagnosed malignant brain tumors in children. A combination of surgery, radiotherapy, and chemotherapy has contributed to improved treatment outcomes, resulting in a 70-80% five-year disease-free survival rate [6]. Still, the mortality rate for patients with medulloblastoma remains significant and recurrence is observed in the clinic, often due to cancer cell resistance to radiation therapy. Moreover, there are significant neurological, cognitive, endocrinological, and social sequelae resulting from current chemotherapy and radiotherapy regimens. In light of this prognosis, significant efforts are underway to develop more effective and less toxic treatments for medulloblastoma patients.

Although little is known about the role of the Eph/ephrin system in medulloblastoma, recent studies have implicated Eph receptors and ephrins as potential players in medulloblastoma tumor progression. For example, overexpression and activation of EphB2, a receptor closely related to EphB1, in medulloblastoma cell lines has been shown to reduce cell adhesion and enhance invasion *in vitro* [7]. Gene expression analyses of the DAOY medulloblastoma cell line further established that EphB1 is highly upregulated in migrating medulloblastoma cells, compared to non-invasive tumor cells at the primary tumor site [8].

The *ATM* gene represents an important component of DNA damage pathways. In our previous studies, we established that mutations in ATM resulted in hypersensitivity to radiation in fibroblasts derived from a patient with mutated ATM [9], and using these cells, we identified molecules regulated by ATM in order to develop targeted radiosensitizers [9]. Furthermore we showed that genetic repair of ATM *via* its expression in the ATM-deficient fibroblast cell line, AT5BIVA, resulted in increased cellular radiation-resistance [10]. Importantly, a greater than 10 fold increase in EphB1 expression was found in the ATM-proficient ATCL8 cells (derived from AT5BIVA) compared to the ATM-deficient AT5BIVA cells [10], suggesting that EphB1 may be responsible, at least in part, for the observed increase in radiation resistance.

Despite these important findings, no additional studies have been reported to date that directly investigate the role of EphB1 in medulloblastoma tumorigenesis. Since EphB1 plays a key function in the development and progression of other cancers, such as glioma, esophageal, colorectal and gastric cancers [11–15], we sought to better define the role of this receptor in medulloblastoma. Using both human medulloblastoma cell lines and genetically engineered mouse models, we investigated the role of EphB1 in medulloblastoma cell growth, migration, and radiosensitization. Herein, we show that knockdown of EphB1 decreased medulloblastoma cell growth and migration, and increased the radiosensitivity of the medulloblastoma cell line *in vitro*. In addition, we developed a new *in vivo* model of EphB1 function in medulloblastoma, by crossing the previously described ND2-SmoA1 preclinical medulloblastoma mouse [16–18] with our *EphB1* knockout mouse model [19, 20]. Using this new model, we show that the genetic loss of *EphB1* results in a significant delay in tumor recurrence following radiotherapy. Collectively, our results are consistent with the hypothesis that upregulation of EphB1 contributes to the aggressive and invasive nature of medulloblastoma. To our knowledge, this study represents the first exploration into the functional role of EphB1 gene in medulloblastoma cell migration, growth, and radiosensitization. Thus, future strategies involving targeted inhibition of EphB1 receptor may hold therapeutic value for the treatment of medulloblastoma.

## RESULTS

### EphB1 is expressed in medulloblastoma tumors

**Figure 1:**
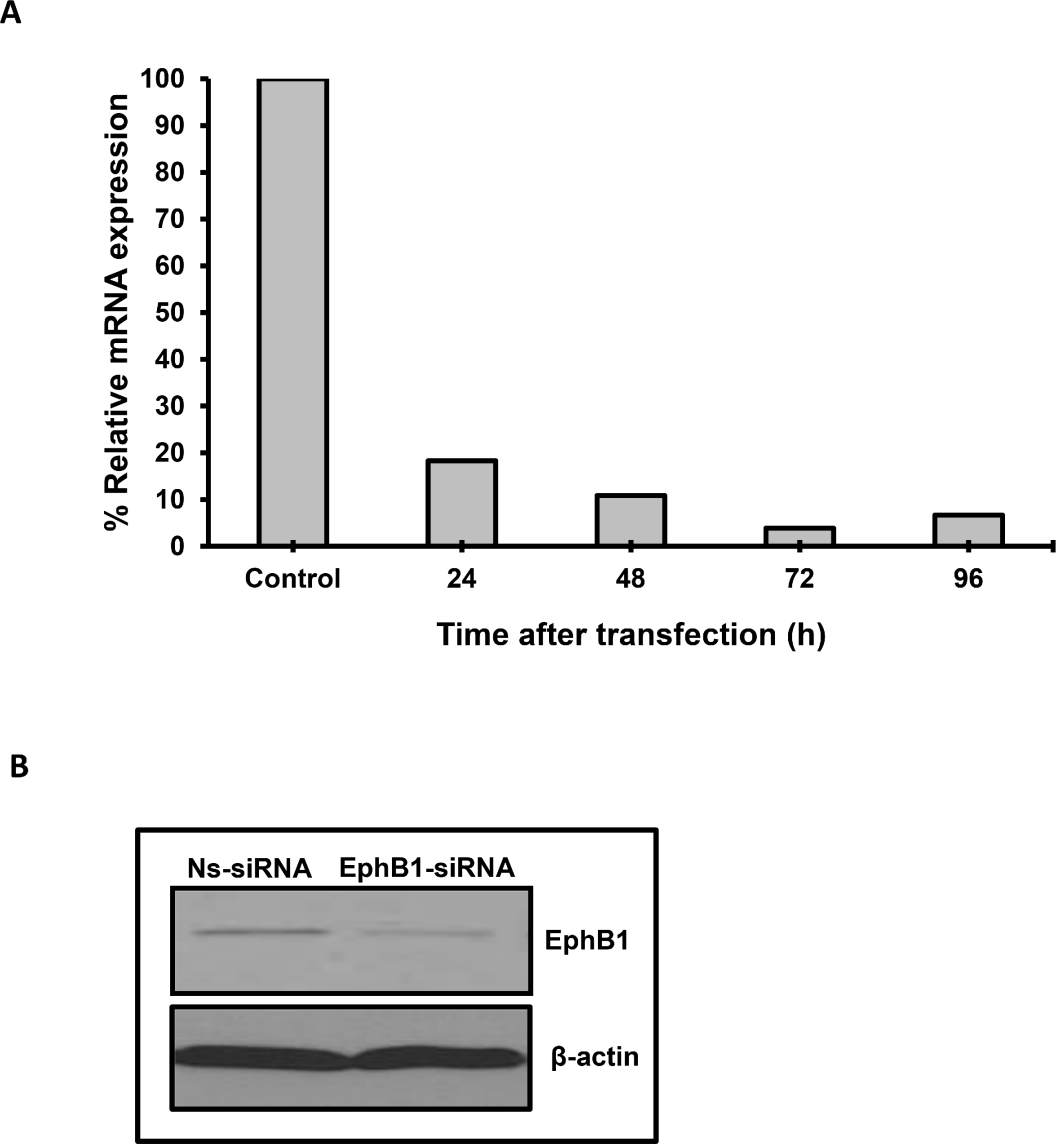
EphB1 is effectively knocked down in DAOY medulloblastoma cells **(A)** EphB1 mRNA level is dramatically reduced upon transfection of DAOY cells with EphB1-siRNA. **(B)** EphB1 expression is detected by western blotting in DAOY cells and is decreased upon transfection with EphB1-targeting siRNA vs. control non-specific siRNA (Ns-siRNA).

The expression of EphB1 receptor varies widely in medulloblastoma [8]. We evaluated the expression of EphB1 in a human medulloblastoma cell line, DAOY, and found EphB1 to be expressed at both the mRNA and protein level (Figure 1A, 1B). To assess the role of EphB1 in medulloblastoma, we next attempted to knockdown EphB1 expression using siRNA approach. DAOY cells were transfected with either EphB1 siRNA or a control, nonspecific siRNA (Ns-siRNA). EphB1 expression was analyzed at the mRNA level at 24, 48, and 72 h post-transfection. We found that EphB1 mRNA levels were reduced to 18% or less by 24 h in the EphB1-knockdown group compared to the control, non-specific siRNA (Ns-siRNA) transfected group, with optimal knockdown efficiency observed at 72 h post-transfection (Figure 1A). Additionally, there was a substantial reduction in the levels of EphB1 protein by western blot analysis of EphB1-knockdown DAOY cells compared to control transfectants (Figure 1B). The results were also replicated in another medulloblastoma cell line, UW228 (Supplementary Figure 1A). Since western blot analysis confirmed an appreciable reduction in EphB1 protein levels, we conducted a series of experiments to determine whether EphB1 downregulation affects cell viability, cell cycle progression, migration, and radiosensitivity.

### Knockdown of EphB1 receptor reduces cell growth and viability

**Figure 2:**
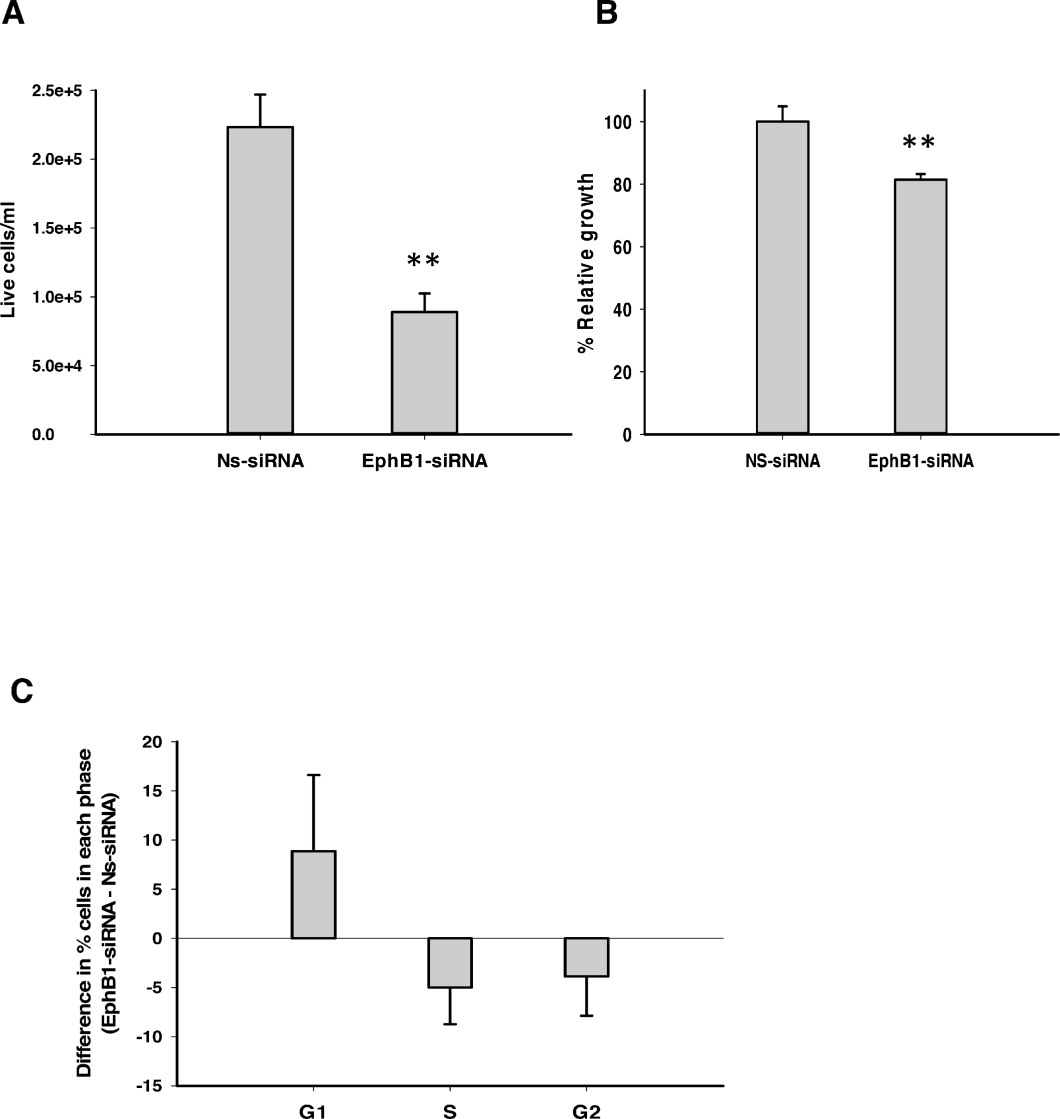
Knockdown of EphB1 reduces medulloblastoma cell viability and cell growth, and alters cell cycle progression **(A)** EphB1 knockdown results in a significant decrease in live cell count as assessed by the trypan blue dye exclusion assay (*n* = 3, *p ≤ 0.01; **p < 0.001). **(B)** Knockdown of EphB1 results in an approximately 26% decrease in cell growth as quantified by MTT assay (*n* = 2, **p < 0.001). **(C)** Knockdown of EphB1 increases percentage of cells in G1 phase (*n* ≥ 2). Data shown are average ± standard error from independent experiments. Normalized values are shown in Figure 2B.

We investigated the effect of EphB1 knockdown on cell growth and viability using total cell count and an MTT assay. DAOY cells transfected with EphB1-targeting siRNA and serum-starved for 24 h were stimulated to grow by addition of serum for 48 h. EphB1-siRNA- transfected cells showed a significant reduction in the number of live cells as determined by trypan blue exclusion, following knockdown of EphB1 receptor (Figure 2A). Additionally, the MTT assays showed a significant reduction in cell growth of about 26% in EphB1-knockdown cells compared to non-specific siRNA (Ns-siRNA) control transfected cells (Figure 2B).

### Knockdown of EphB1 receptor inhibits cell cycle progression

We next sought to determine whether EphB1 knockdown affects cell cycle progression. DAOY cells were transfected, serum-starved and then stimulated by addition of serum as above. Cell cycle distribution was analyzed by flow cytometry as previously described [21–23]. A higher percentage of cells in the G1 phase were observed in EphB1-knockdown cells compared to the non-specific siRNA (Ns-siRNA) transfected cells (Figure 2C). Similar results were obtained with another well-studied medulloblastoma cell line, UW228, where EphB1 knockdown showed an approximately 11% increase in the percentage of cells in the G1 phase as compared to the control non-specific siRNA (Ns-siRNA) transfected group (Supplementary Figure 1B).

### EphB1 knockdown modulates cell cycle, cell proliferation, and survival proteins

**Figure 3:**
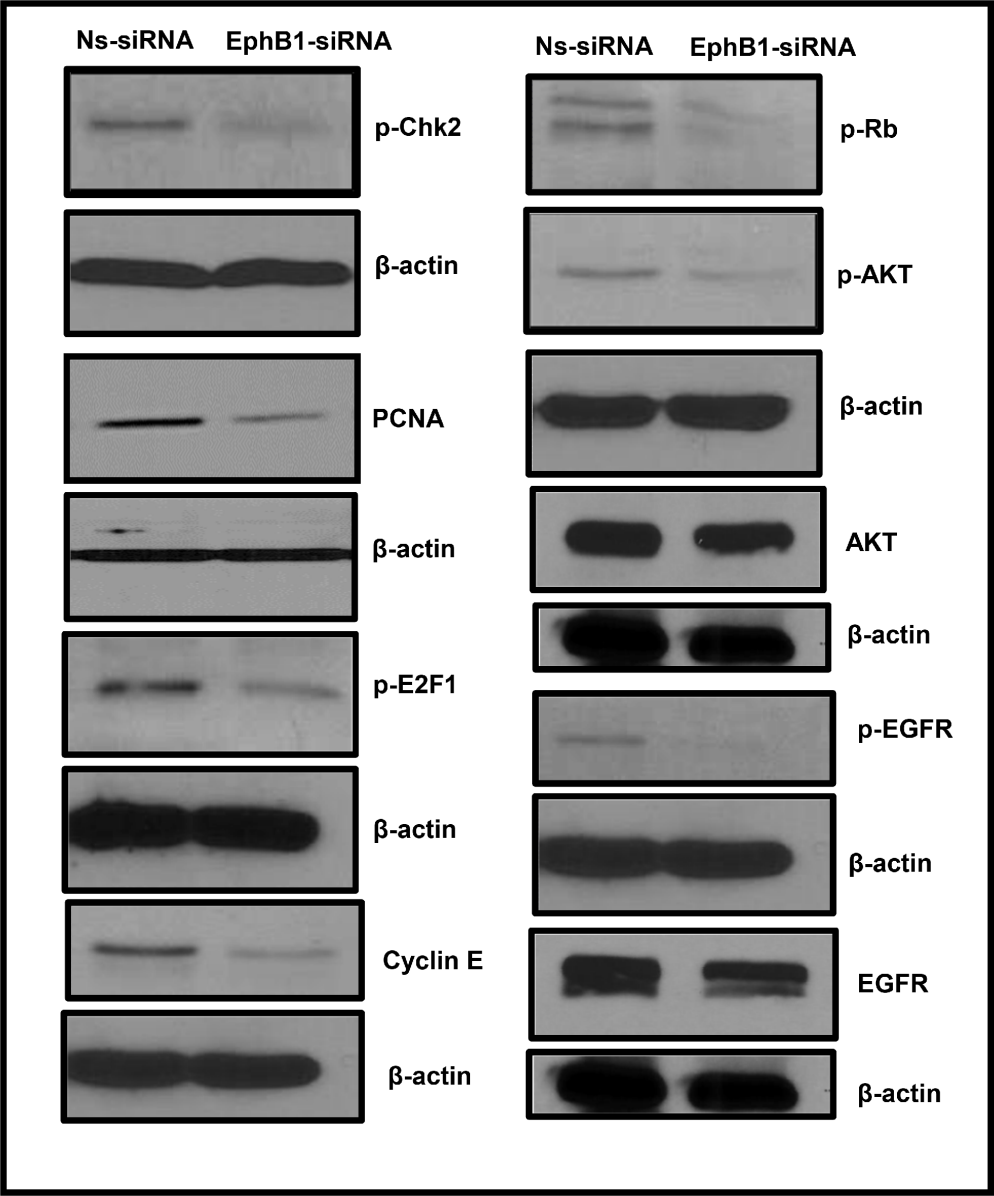
EphB1 knockdown modulates the expression of cell cycle, proliferation, cell survival proteins Changes were observed in the expression of p-Chk-2, PCNA, p-E2F1, p-Rb, p-AKT, AKT, cyclin E, p-EGFR, and EGFR proteins following EphB1 receptor knockdown compared to the non-specific siRNA (Ns-siRNA) treatment in DAOY cells.

We next investigated the mechanism underlying the role of EphB1 in DAOY cells by evaluating the expression of proteins known to play a role in cell cycle progression and cell survival. We found that EphB1 downregulation resulted in a decrease in the levels of cyclin E, phosphorylated E2F1 (p-E2F1), phosphorylated Chk2 (p-Chk2), and phosphorylated Rb (p-Rb) (Figure 3). We also evaluated the levels of PCNA, a proliferation marker, and phosphorylated Akt, a protein whose phosphorylation promotes cell survival. We found that levels of both proteins decreased following EphB1 knockdown (Figure 3). Total AKT levels were also decreased in EphB1-siRNA treated cells (Figure 3). In view of the fact that Eph receptors are known to functionally interact with other tyrosine kinase receptors to promote malignant cell behavior [24], we also analyzed the levels of phosphorylated epidermal growth factor receptor (p-EGFR) and total EGFR and found that p-EGFR and EGFR levels were reduced in the EphB1 knockdown cells (Figure 3).

### Knockdown of EphB1 receptor decreases migration

As EphB1 has been reported to promote migration and chemotaxis in several systems [25, 26], we next investigated the impact of EphB1 knockdown on medulloblastoma cells in an electrical impedance-based migration assay. We transiently transfected DAOY cells with the EphB1-targeting siRNA as described above and evaluated the effects of EphB1 knockdown on cell migration post-transfection.

**Figure 4:**
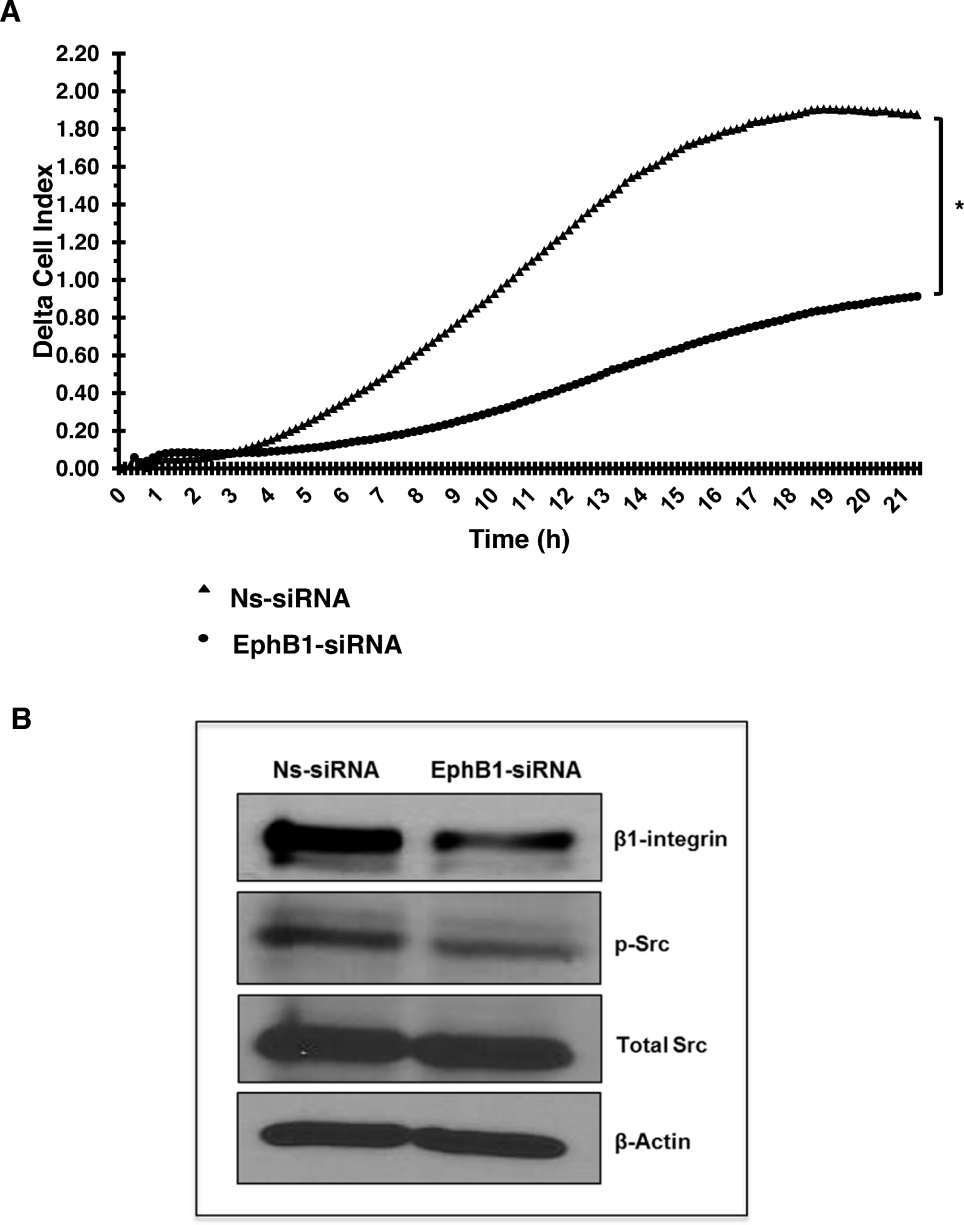
Knockdown of EphB1 receptor reduces medulloblastoma cell migration **(A)** A transwell chamber using electrical impedance measurements for cell migration was used. Cell index values represent changes in electrical impedance across the membrane separating the upper and lower chambers, and correlates directly to the number of cells that have migrated into the lower chamber. Background migration was subtracted to obtain delta cell index values. Knockdown of EphB1 decreases the migratory response on average by 51% (*p < 0.01) compared to control non-specific siRNA (Ns-siRNA) transfection. **(B)** Knockdown of EphB1 results in decreased expression of β1-integrin and p-Src.

Compared to control transfectants, knockdown of EphB1 resulted in a 51% decrease in migration after 21 h (Figure 4A). We further investigated the role of EphB1 on migration by evaluating the expression of molecules involved in cell adhesion and migration by western blot analysis. EphB1 knockdown resulted in decreased expression of β1-integrin and decreased levels of phosphorylated Src while levels of total Src remained constant (Figure 4B).

### Knockdown of EphB1 enhances radiosensitization in ATM-proficient fibroblast cells

**Figure 5:**
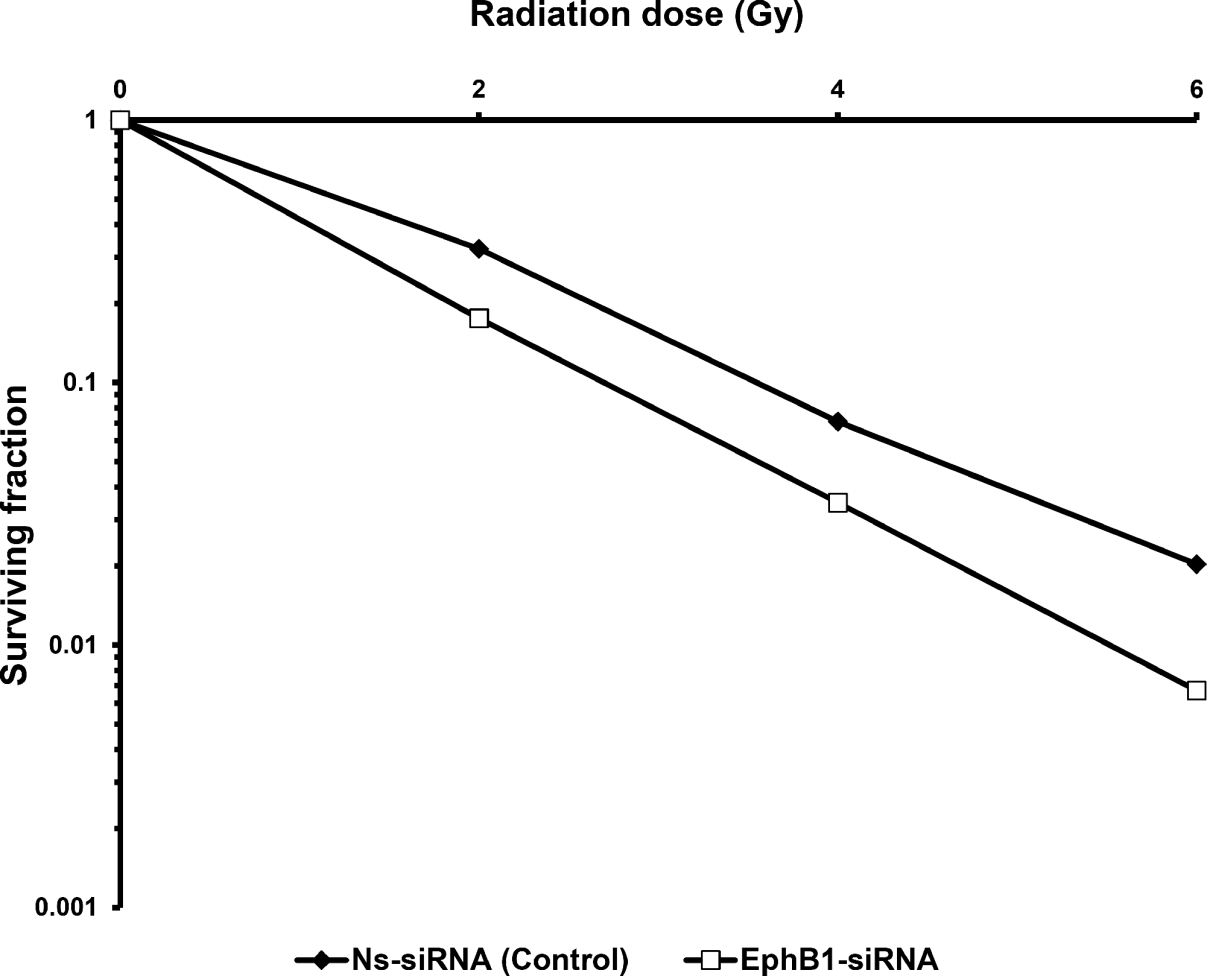
Knockdown of EphB1 receptor sensitizes fibroblasts to ionizing radiation Survival curves from clonogenic assays comparing ATM-proficient ATCL8 cells transfected with EphB1-targeting siRNA to control transfectants show that EphB1 targeting reduces cell survival.

Savitsky *et al*. have found ATM to be a critical regulatory molecule that recognizes DNA damage and activates cellular signaling pathways to protect the genome [27]. Mutations in ATM lead to hypersensitivity to ionizing radiation [28]. To investigate whether EphB1 plays a role in radiation resistance, we knocked down EphB1 in ATM-proficient ATCL8 cells, exposed the cells to a single dose of 2, 4, or 6 Gy of radiation, and performed clonogenic survival assays. Decreased EphB1 expression was accompanied by a significant increase in sensitivity of ATCL8 cells to irradiation (Figure 5). The dose enhancement ratio (D_0_ value) for non-specific siRNA (Ns-siRNA) group was found to be 1.541 in the clonogenic survival assay. For EphB1-siRNA treated group, D_0_ value was reduced to 1.201.

### Knockdown of EphB1 receptor sensitizes medulloblastoma cells to ionizing radiation

**Figure 6:**
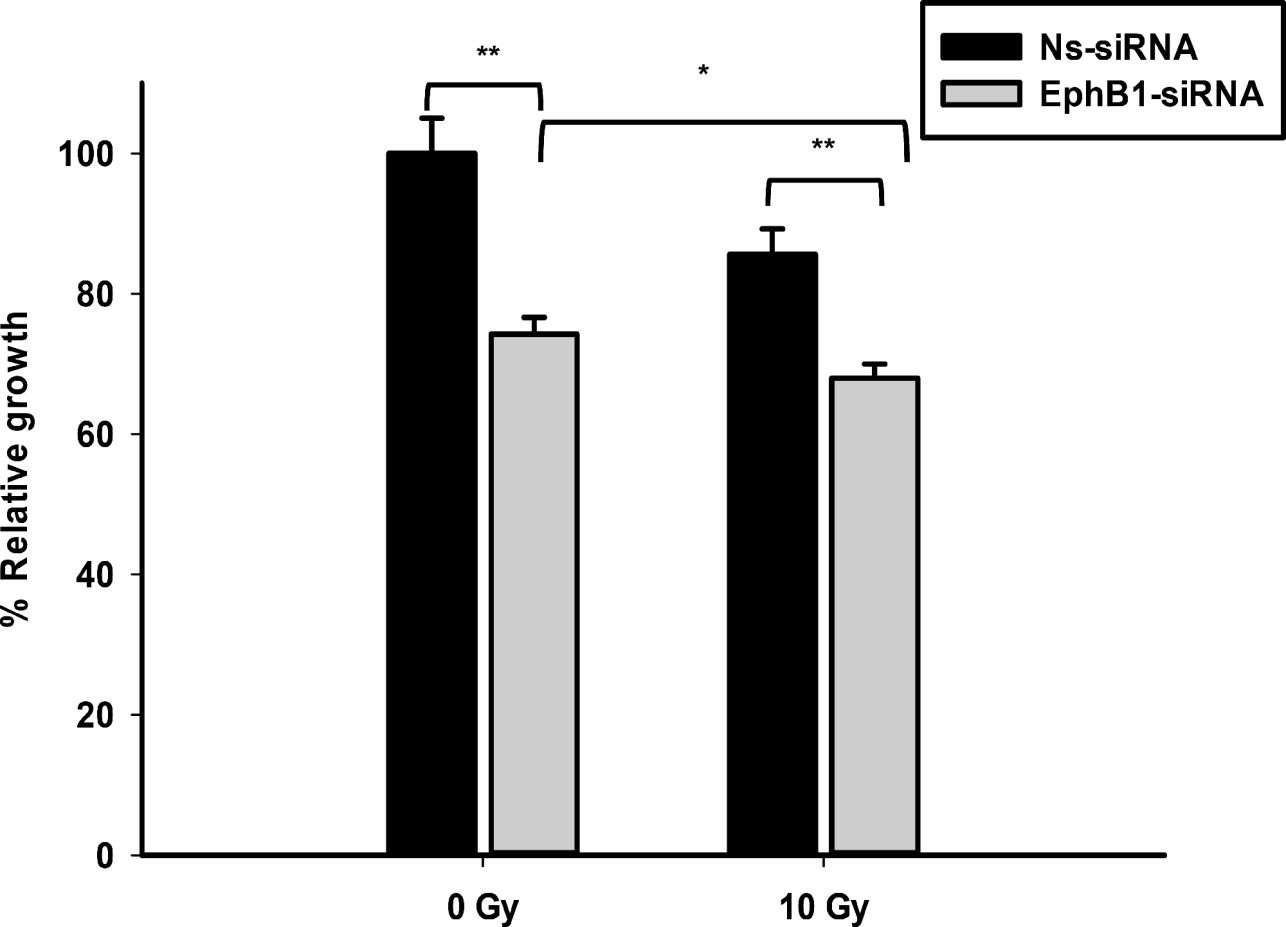
Knockdown of EphB1 receptor sensitizes DAOY cells to ionizing radiation DAOY cells transiently transfected with either non-specific siRNA (Ns-siRNA) or EphB1-targeting siRNA were irradiated at 24 h post-transfection with 10 Gy. At 96 h post-transfection, MTT reagent was added to samples and optical density measured after 24 h incubation. Data shown are normalized values and represent average ± standard error with **p < 0.001, *p < 0.05.

**Figure 7:**
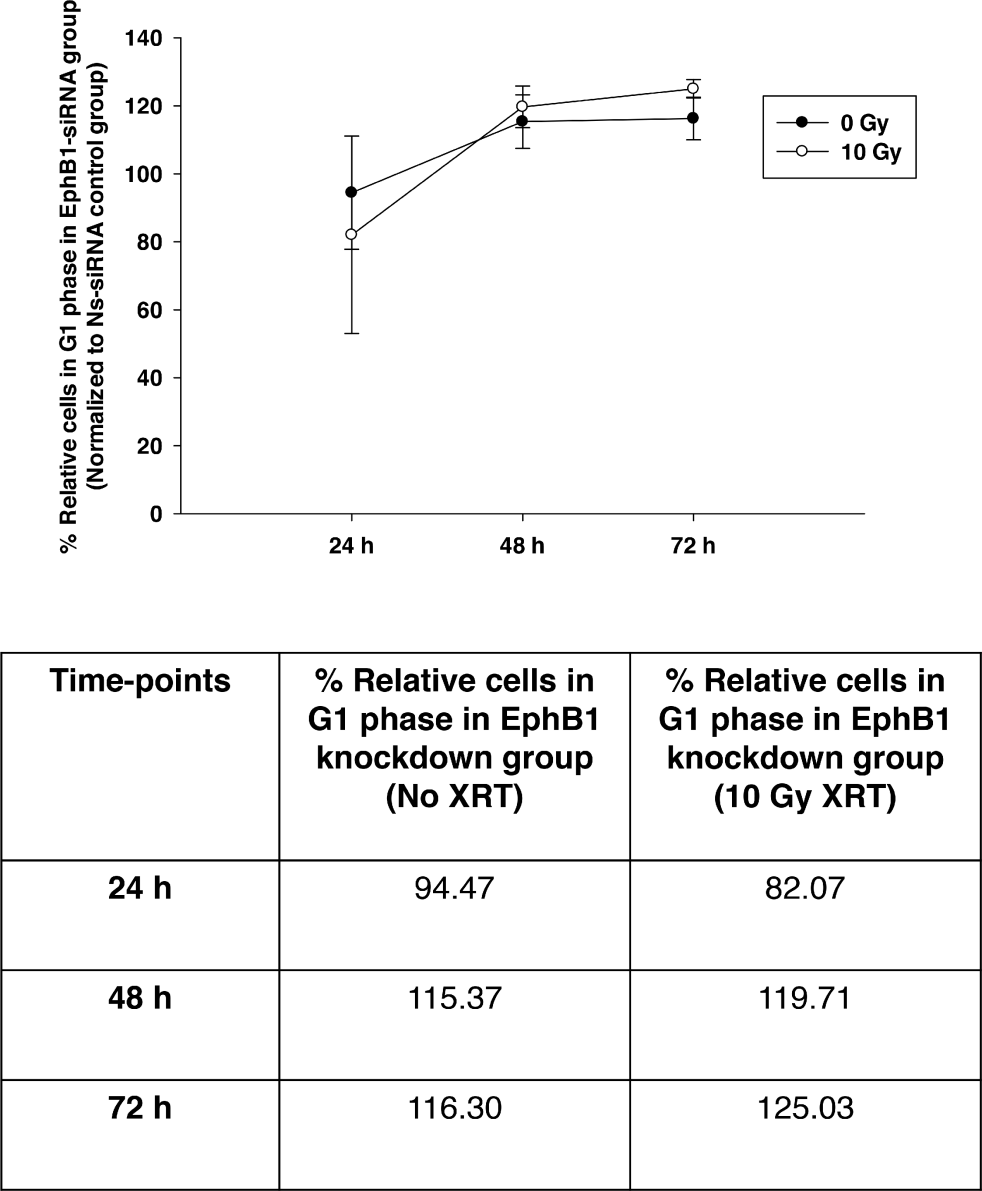
Knockdown of EphB1 receptor enhances the percentage of cells in G1 phase in irradiated DAOY cells DAOY cells were transiently transfected with non-specific siRNA (Ns-siRNA) or EphB1-targeting siRNA and irradiated with 10 Gy at 24 h post-transfection. At 24, 48, and 72 h post-radiation, cell cycle profiles were assessed by flow cytometry. Data points represent the percentage of G1 cells in EphB1-siRNA treated group (normalized to Ns-siRNA treated group) in the absence and presence of ionizing radiation. Data shown are average ± standard error for *n* ≥ 2 independent experiments. The percentage of EphB1-siRNA treated cells in G1 phase of cell cycle at different time-points are indicated in the tabular format.

To investigate whether EphB1 also plays a role in radiation sensitivity, we compared the responses of the EphB1-knockdown vs. control siRNA in DAOY cells. Using a single dose of 10 Gy of ionizing radiation, cell growth was determined at 96 h post transfection using an MTT assay. The percentage of cell growth was reduced by approximately 26% in the EphB1 knockdown cells vs. the non-specific siRNA (Ns-siRNA)-treated DAOY cells (Figure 6). Furthermore, cell growth was reduced by approximately 32% in the irradiated EphB1 knockdown cells (Figure 6). In addition, analysis of cell cycle profiles by flow cytometry in DAOY cells indicated that knockdown of EphB1 expression enhanced the percentage of cells in G1 phase in both non-irradiated and irradiated (10 Gy) samples at both 48 h and 72 h post-irradiation, with a more dramatic effect observed at 72 h (Figure 7).

### Knockdown of EphB1 receptor does not radiosensitize medulloblastoma cells by apoptosis

To further investigate the mechanism by which EphB1 knockdown enhances cellular radiosensitization, we evaluated the role of EphB1 downregulation in triggering apoptosis in DAOY cells. Following EphB1 receptor knockdown and exposure of cells to 10 Gy ionizing radiation, we used Annexin V/propidium iodide (PI) staining to detect a subpopulation of cells undergoing apoptosis. Our data indicate that neither knockdown of EphB1 nor ionizing radiation exposure resulted in a significant increase in the percentage of cells undergoing apoptosis compared to the non-specific siRNA (Ns-siRNA) treatment (Supplementary Figure 2). We further determined the expression of classical apoptotic markers following treatment of DAOY cells with EphB1-siRNA and control Ns-siRNA. Our results suggest that EphB1 knockdown does not induce PARP cleavage in DAOY cells (Supplementary Figure 3). Further, we did not observe any changes in the levels of pro- and anti-apoptotic markers such as Bax and Bcl-XL (Supplementary Figure 3) in the EphB1-siRNA vs. control Ns-siRNA treated cells.

### Loss of EphB1 expression enhances radiosensitization *in vivo*

**Figure 8:**
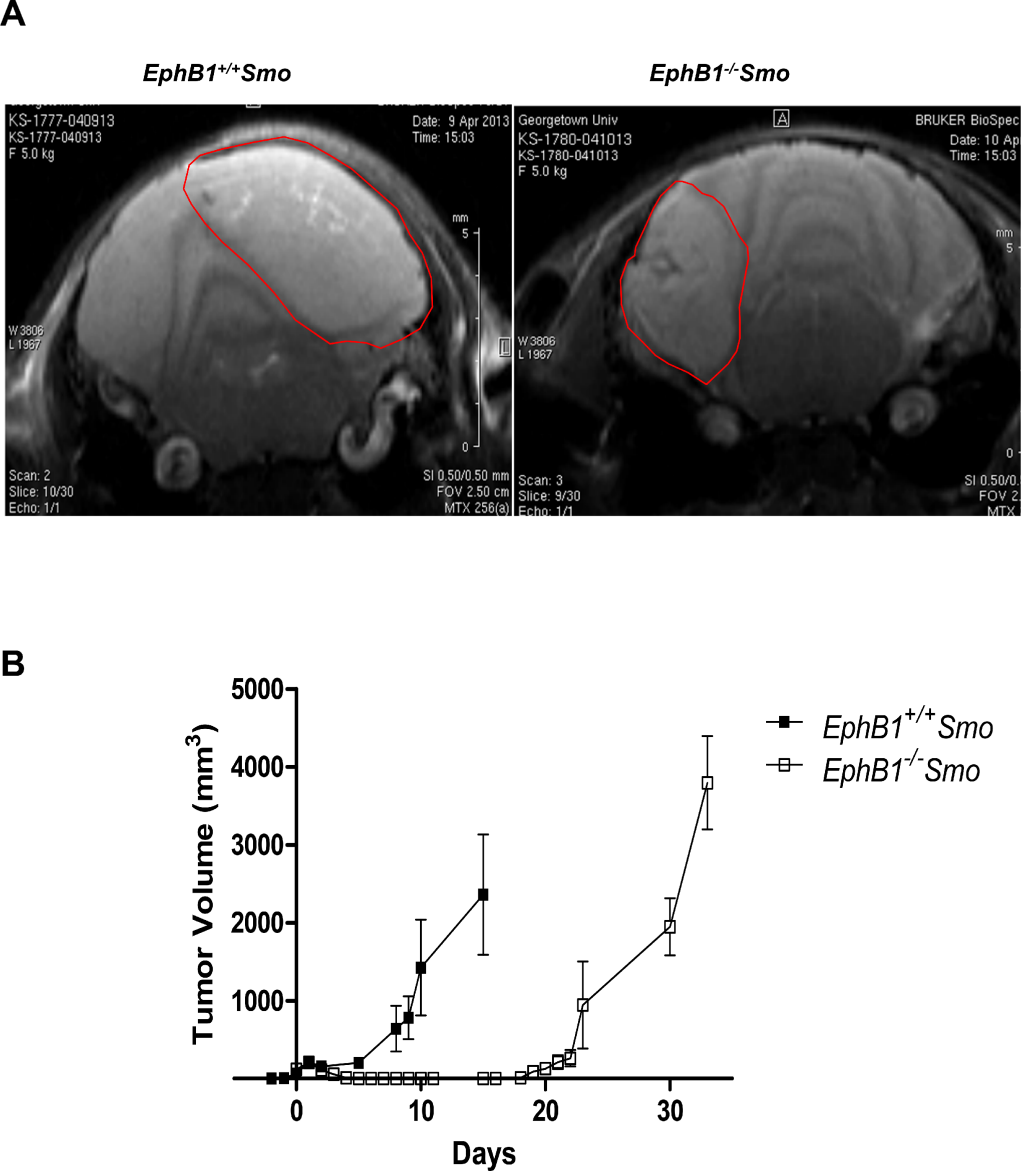
Tumors harvested from EphB1-deficient mice demonstrate enhanced tumor growth delay after irradiation **(A)** MRI imaging shows the development of cerebellar medulloblastomas in genetically engineered medulloblastoma mouse model. *EphB1^−/−^Smo* mice and their *EphB1^+/+^Smo* littermate controls (age-matched and gender-matched) were followed by serial MRI imaging for target delineation from postnatal day 28 until onset of disease or euthanization (*n* = 44). Representative images are shown for littermate *EphB1^+/+^Smo* and *EphB1^−/−^Smo* pairs. **(B)** Tumors harvested from *EphB1^−/−^Smo* mice and their littermate *EphB1^+/+^Smo* controls were transplanted subcutaneously into the mid-dorsal surface of the mouse just rostral to the tail. Tumor masses harvested from *EphB1^−/−^Smo* mice exhibited a three-fold delay in tumor recurrence compared to tumor masses harvested from littermate EphB1^+/+^Smo controls after fractionated ionizing radiation (5 Gy/day × 4 doses) treatment (*p* < 0.001).

To examine the possible effects of EphB1 on radiosensitivity *in vivo*, we generated a genetically engineered EphB1 knockout mouse medulloblastoma model (*EphB1^−/−^Smo*) by crossing the transgenic ND2-SmoA1 medulloblastoma line [17] with our mice deleted of *EphB1* [19, 20]. Gender-matched littermate *EphB1^+/+^Smo* mice were used as controls. Tumor development was assessed by MRI as previously described [16, 18] from postnatal day 23 to 12 months of age, or until the mice began displaying symptoms such as ataxia, decreased motor and coordination functions, megalocephaly, hunching, and weight loss. Longitudinal MRI imaging was conducted to delineate the tumors (Figure 8A). A total of 44 mice were imaged and 128 MRI images were obtained. Once the tumors reached 200-300 mm^3^, the tissues were harvested, minced, and equivalent numbers of tumor cells were mixed with matrigel and subcutaneously implanted in the mid-dorsal region of the nude mice just rostral to the tail. Once palpable tumors formed, the mice were randomized into four different groups. One group each from the *EphB1^−/−^Smo* and *EphB1^+/+^Smo* cohorts were used as non-irradiated controls while the other two groups of mice were treated with fractionated ionizing radiation (5 Gy/day × 4 doses). Tumor volumetric growth kinetics before, during, and after radiation treatment were quantified using caliper measurements.

While the loss of EphB1 did not significantly affect tumor growth in the control animals (data not shown), allografted tumors from *EphB1^−/−^Smo* mice exhibited a three-fold delay in tumor recurrence after fractionated ionizing radiation compared to allografts harvested from *EphB1^+/+^Smo* controls (Figure 8B).

## DISCUSSION

Aberrant Eph/ephrin signaling has been implicated in a wide variety of human cancers including medulloblastoma [29]. Accumulating evidence suggests a role of Eph receptors and their ligands in tumor cell survival, invasion, and metastasis [30, 31]. In the present study, we investigated the functions and mechanisms underlying the effects of EphB1 receptor knockdown on cell growth, migration, and radioresistance in medulloblastoma. Our findings indicate that EphB1 downregulation reduces migration and enhances cellular sensitization to radiation therapy by increasing the percentage of cells in the G1 phase of the cell cycle ultimately decreasing medulloblastoma cell growth and viability. Our results are in agreement with published reports suggesting a role of EphB1 in medulloblastoma tumorigenesis [8, 32]. Morrison *et al*. recently showed that EphB1 transcript levels are significantly upregulated in migrating medulloblastoma cells as compared to non-migrating medulloblastoma cells [8]. EphB1 transcript levels were also found to be significantly upregulated in medulloblastoma tumor spheres with high self-renewing ability compared to tumor spheres with low self-renewing ability [8].

Eph receptor tyrosine kinases and their ephrin ligands have emerged as important players in medulloblastoma tumorigenesis [33]. Infact, microarray data analysis from independent datasets confirm the presence of EphB1 receptor in different clinical medulloblastoma tumor subtypes [34–36]. In a recent study by McKinney *et al*., it was reported that EphB1 receptor is expressed in different subgroups of medulloblastoma tumors examined [33]. Since, EphB1 is widely expressed in medulloblastoma, we analyzed the expression of EphB1 in human medulloblastoma cell lines DAOY and UW228. These cell lines closely resemble different medulloblastoma subtypes [37]. Our data suggest that both these lines express considerable levels of EphB1 receptor and thus serve as appropriate model systems to study the effects of EphB1 knockdown on radiosensitization in medulloblastoma.

**Figure 9:**
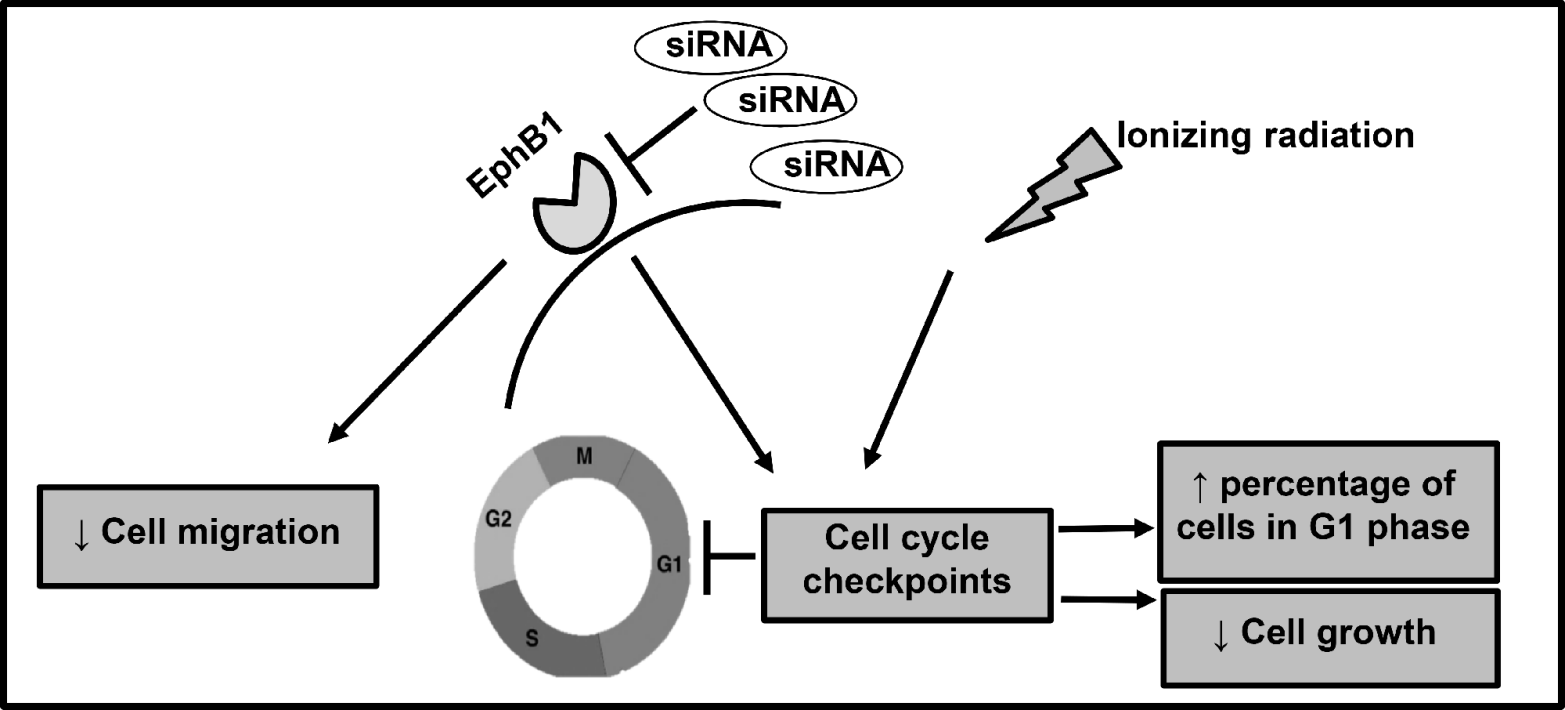
Proposed hypothetical model EphB1 receptor knockdown radiosensitizes medulloblastoma cells and decreases cell growth, and enhances the percentage of cells in G1 phase of cell cycle. Additionally, siRNA-mediated EphB1 knockdown reduces cell migration.

The Eph/ephrin system has been reported to play a key role in cancer cell growth and proliferation. Previous studies report anti-proliferative effects of Eph receptors, mediated primarily by suppression of the ERK/MAPK pathway [29, 38–41]. In contrast, recent findings indicate that some Eph receptors promote cell proliferation, for example in colorectal cancer and in the developing nervous system [30, 42–44]. During development, lack of EphB1 in a mouse model has been reported to significantly reduce the number of neural progenitors in the hippocampus, as well as disrupt the proper migration and organization of neural progenitors [42]. Consistent with these findings, we report that EphB1 knockdown decreases medulloblastoma cell growth and viability. Furthermore, our data provide mechanistic insights into how EphB1 downregulation affects medulloblastoma cell growth and survival (Figure 9). The reduction in the growth and viability of EphB1 knockdown DAOY cells could be explained in part by the decreased expression of cyclin E, a master regulator of cell cycle progression, which in turn results in decreased levels of phosphorylated Rb and E2F1 proteins and also affects the activation of a checkpoint kinase, Chk2, resulting in cell cycle arrest. In addition to playing a regulatory role, overexpression of cyclin E has been linked to tumorigenesis in different cancers [45]. In view of this, inhibition of cyclin E following EphB1 knockdown likely plays a role in the observed inhibition of medulloblastoma tumor growth. Importantly, the decreased ability of DAOY cells to progress from G1 to S phase following EphB1 knockdown, could ultimately be responsible for inhibiting their growth, proliferation, and survival as indicated by reduced expression of PCNA and decreased levels of phosphorylated Akt, and total AKT. Thus, our data clearly underscore the relevance of EphB1 knockdown in inhibiting medulloblastoma cell growth by affecting cell cycle, proliferation, and cell survival pathways. Cross-talk between Eph receptors and other receptor tyrosine kinases has been reported to contribute to malignant cell behavior [24, 31, 46]. The role of EGFR signaling in tumorigenesis is well established [47]. Our results show an appreciable decrease in the levels of phosphorylated EGFR and total EGFR following EphB1-specific siRNA treatment in DAOY cells, suggesting that the EphB1 receptor might be contributing to the metastatic behavior of medulloblastoma cells by functionally interacting with EGFR. This could be relevant in view of the fact that many human cancers have elevated expression of EGFR along with Eph receptors. Thus, targeted therapeutics developed against Eph receptors could potentially be a viable and effective treatment option against EGFR-positive cancers as well.

The role of the Eph/ephrins in cell migration, invasion, and metastasis has been well documented in other tumor models [29]. For instance, downregulation of EphB1 has been shown to reduce migration of neural progenitors in the hippocampus during development [42, 48]. In contrast, enhanced EphB1 signaling has been shown to decrease migration and invasion in glioma cell lines [12]. These different results could be partly explained by the fact that the effect of Eph receptors is highly context-dependent and can vary across different cancer types [29]. Our results indicate that EphB1 knockdown significantly decreases the migratory ability of medulloblastoma cells in culture. This is consistent with previous reports suggesting that EphB1 acts as a pro-migration receptor [25, 26, 49]. Furthermore, our results suggest that downregulation of EphB1 impedes cell migration *via* alterations in the level of β1-integrin and phosphorylated Src. This could be a potential mechanism by which EphB1 contributes to medulloblastoma metastasis. This is in accordance with the study published by Huynh-Do *et al*., wherein the EphB1 ligand, ephrinB1, was found to mediate cell migration through an integrin-dependent pathway [50]. In another study, it was shown that EphB1 regulates cell migration and chemotaxis *via* stimulation of c-Src activity [26]. Our findings also emphasize the potent role of EphB1 in cancer cell migration.

ATM acts as a key mediator of DNA damage response pathways [27]. It has been reported that ATM regulates EphB1 expression [10]. This was validated in fibroblasts, where supplementing wild-type ATM expression in ATM-deficient fibroblasts significantly upregulated EphB1 expression and altered the radiosensitization profile of the cells [10]. In our study, we report that upon EphB1 knockdown, ATM-proficient cells became more sensitive to radiation, thus decreasing survival in clonogenic assays. Other members of the Eph receptor and ephrin families, including EphA2, EphB4, ephrin A1, and ephrin A3, have previously been implicated in radioresistance in different tumor models [51–54]. In line with these studies, we found that siRNA-mediated downregulation of EphB1 receptor modulates the cellular sensitivity of medulloblastoma cells to ionizing radiation *in vitro*. This was evident in cell cycle analysis where EphB1 knockdown followed by ionizing radiation resulted in an increase in the percentage of DAOY cells in G1 phase of the cell cycle. Given that early G1 is a radioresistant phase of the cell cycle [55], we believe that knockdown of EphB1 forced cells to arrest in late G1 phase, when cells are relatively more sensitive to radiation [55–57]. This in turn affected tumor growth and progression as validated in our experimental model systems. Cells that have a long cycling time, a peak of resistance observed in early G1 phase of cell cycle is followed by a radiosensitive phase toward the end of G1 phase [55]. Other research groups have also shown late G1 cell cycle arrest following treatment with anti-cancer agents and radiation [56, 57]. However, upon further investigating into the mechanism of radiosensitization, we did not observe any significant increase in apoptosis or changes in the levels of apoptotic markers upon EphB1 knockdown. We also performed senescence assays to evaluate the role of EphB1 knockdown on cell senescence. However, we did not see any measurable effect on senescence in our study upon EphB1 receptor downregulation (data not shown). This suggests the involvement of other pathways such as DNA damage repair that might be playing a role in EphB1 knockdown mediated radiosensitization in medulloblastoma cells. Studies are currently underway to support this notion.

Importantly, our data indicate that EphB1 loss enhances radiation sensitivity *in vivo* as observed in the genetically modified mouse model of medulloblastoma. The transgenic mouse model used in this study has high rate of tumor incidence and it closely resembles the pathological features evident in the human medulloblastoma tumors thus, making it as an important preclinical model system to predict the treatment response. In the present study, we report that the loss of EphB1 did not significantly alter tumor growth in the non-radiated cohort (data not shown). However, tumors that were implanted from *EphB1^−/−^Smo* mice exhibited a three-fold delay in tumor recurrence after fractionated ionizing radiation compared to allografts harvested from *EphB1^+/+^Smo* controls. This is an important finding providing further evidence that EphB1 loss plays a crucial role in enhancing the sensitivity to radiation therapy in medulloblastoma.

In summary, we demonstrate that knockdown of EphB1 receptor decreases medulloblastoma cell growth and migration, increases the number of cells in the G1 phase, and enhances cellular sensitization to ionizing radiation. The influence of EphB1 suppression on medulloblastoma tumorigenesis makes EphB1 a potential candidate for the development of targeted therapies. Thus, therapeutic interventions directed at the EphB1 receptor hold potential in the context of medulloblastoma and other human malignancies.

## MATERIALS AND METHODS

### Cell culture and transfection

The human medulloblastoma cell lines DAOY and UW228 were obtained from the American Type Culture Collection (ATCC, Rockville, MD, USA). The ATM-deficient human fibroblast cell line AT5BIVA was obtained from the National Institute of General Medical Sciences (NIGMS). The ATM-proficient human fibroblast cell line ATCL8 was established by transfecting AT5BIVA cells with the wild-type, full-length ATM gene in a pcDNA expression vector. AT5BIVA cells were maintained in modified Eagle's medium with 20% fetal bovine serum, 100 U/penicillin, and 100 pg/mL streptomycin. DAOY and UW228 cells were maintained in Dulbecco's Modified Eagle's Medium (DMEM), supplemented with 10% fetal bovine serum, 1% penicillin-streptomycin, and 1% L-glutamine (Life Sciences) at 37°C and 5% CO_2_. For transfection experiments, sub-confluent DAOY and UW228 cells were transfected in serum-free DMEM using TransIT-TKO Transfection Reagent (Mirus, Madison, WI, USA), according to the manufacturer's instructions. Short interfering RNAs (siRNA) specific for human *EphB1* and the non-specific control RNAi were obtained from Invitrogen (Carlsbad, CA, USA). Briefly, cells were transfected using 10 μL TransIT-TKO for a final concentration of 25 nM siRNA for migration studies, or 50 nM siRNA for proliferation studies.

### Quantitative real-time PCR

Total RNA was extracted from adherent cells using the RNeasy Kit (Qiagen, Valencia, CA, USA) and reverse-transcribed using the High Capacity cDNA Reverse Transcription Kit (Applied Biosystems) according to the manufacturer's instructions. To assess the expression levels of EphB1, quantitative real-time PCR (qRT-PCR) was performed using the TaqMan Gene Expression Assay (Applied Biosystems) on the Applied Biosystems 7900HT Fast Real-Time PCR System, using fast mode. Expression of β-actin was used to control for RNA loading. Data were analyzed using the comparative cycle threshold (CT) method, as described previously [58].

### Whole cell lysate collection

DAOY cells were homogenized in RIPA lysis buffer (Millipore, MA, USA), containing protease inhibitor cocktail (Thermo Fisher Scientific Inc., IL, USA) and phosphatase inhibitor (Sigma, MO, USA) on ice for 30 min. The homogenate was centrifuged at 4°C at 13,000 rpm for 20 min, and the supernatant was collected for analysis of protein expression by western blotting. Protein was quantified using the BCA Protein Assay kit (Thermo Fisher Scientific Inc., IL, USA).

### Western blotting and antibodies

Protein extracts (20–30 μg) were loaded onto 10% SDS-PAGE gels. Electrophoresis, blocking, probing, and visualization of proteins were conducted as described [59, 60]. Blots were probed overnight at 4°C with the respective antibodies. All primary antibodies (anti-EphB1, anti-cyclin E, anti-p-Akt, AKT, anti-p-EGFR, anti-p-Chk2, p-Src, Src, p-Rb, PARP, Bax, Bcl-XL, and anti-β-actin) were obtained from Cell Signaling Technology (Danvers, MA, USA) except anti-PCNA (BD Biosciences, San Jose, CA, USA), anti-p-E2F1, EGFR (Santa Cruz Biotechnology, Dallas, TX, USA), and anti-β1-integrin (Abcam, Cambridge, MA, USA). Horseradish peroxidase (HRP)-conjugated secondary antibodies were obtained from Sigma (St. Louis, MO, USA).

### MTT assay

DAOY cells were seeded at a density of 50,000 cells per well in 24-well plates and maintained in DMEM supplemented with 10% FBS, 1% penicillin-streptomycin, and 1% L-glutamine for 24 h prior to transfection with siRNA. Subconfluent cells were transfected in serum-free DMEM containing 1% penicillin-streptomycin and 1% L-glutamine. At 24 h after transfection, medium was exchanged for 10% DMEM supplemented with 1% penicillin-streptomycin, and 1% L-glutamine to stimulate proliferation. The MTT (3-(4, 5-dimethylthiazol-2-yl)-2, 5-diphenyltetrazolium bromide) assay was performed using the Cell Proliferation Kit I (MTT) (Roche Applied Science, Indianapolis, IN, USA). After 48 h stimulation, 50 μL MTT was added to each well and cells were incubated for 4 h at 37°C. 250 μL SDS (sodium dodecyl sulfate) was added to dissolve formazan crystals. Cells were incubated for another 24 h at 37°C, and optical density was measured on a microplate reader at 550 nm, with a reference filter wavelength of 655 nm. Optical density measurements were compared across control and EphB1-knockdown samples using Student's *t* test.

### Trypan blue dye exclusion

DAOY cells were seeded at a density of 200,000 cells per well in 6-well plates and maintained in DMEM supplemented with 10% FBS, 1% penicillin-streptomycin, and 1% L-glutamine for 24 h before transfection. Cells were transfected in serum-free DMEM, with 1% penicillin-streptomycin and 1% L-glutamine. At 24 h after transfection, medium was exchanged for 10% DMEM supplemented with 1% penicillin-streptomycin, and 1% L-glutamine to stimulate proliferation. After 48 h stimulation, cells were collected by trypsinization. To each 10 μL aliquot of cells, 10 μL of trypan blue dye was added. Cell counting was performed using the Countess Automated Cell Counter (Life Technologies, NY, USA).

### Flow cytometry

DAOY and UW228 cells were seeded at a density of 200,000 cells per well and 150,000 cells per well respectively in 6-well plates. Cells were maintained in 10% DMEM supplemented with 1% penicillin-streptomycin, and 1% L-glutamine for 24 h before transfection. Cells were transfected using 25 nM (UW228 cells) or 50 nM (DAOY cells) siRNA in serum-free DMEM, with 1% penicillin-streptomycin and 1% L-glutamine. At 24 h after transfection, medium was exchanged for 5% or 10% DMEM supplemented with 1% penicillin-streptomycin, and 1% L-glutamine to stimulate proliferation. After 48 h stimulation, dead cells were collected by aspirating medium and viable cells were collected by trypsinization. Samples were washed twice in ice-cold PBS, fixed in 70% ethanol, stained with propidium iodide, and analyzed for cell cycle distribution by fluorescence-activated cell sorting. Data from at least three independent experiments were analyzed by Student's *t*-test.

### Electrical impedance-based boyden chamber assay

The xCELLigence RTCA DP Analyzer (Roche, CA) was used to monitor migration in a CIM-16 plate (ACEA Biosciences). The CIM-16 plate is a modified Boyden chamber in which the porous membrane separating the double-chambered well is coated with gold microelectrodes. In this system, cells migrating from the upper chamber into the lower chamber adhere to these microelectrodes, increasing the electrical impedance across this membrane. Electrical impedance is measured as a “cell index,” which directly correlates to the number of cells that have migrated into the lower chamber and adhered to the underside of the membrane. Background measurement was taken by adding cell-free, serum-free DMEM to the upper and lower chambers of each well. The plate was incubated at 37°C for 1 h prior to measuring background electrical impedance for each well. At 48 h post-transfection, cells were starved for 7 h in serum-free DMEM supplemented with 1% penicillin-streptomycin and 1% L-glutamine, prior to collection by trypsinization. Cells were resuspended in serum-free DMEM with 1% penicillin-streptomycin and 1% L-glutamine and seeded in the upper chamber at a density of 50,000 cells per well. Migration was monitored for 24 h. Delta cell index values were calculated by subtracting cell index values in the absence of a serum gradient from cell index values in the presence of a serum gradient. Cell index values between different treatment groups were determined at 30 minute-intervals between 4 and 21 h after the start of the experiment. Statistical analysis was performed using Student's *t* test.

### Clonogenic survival assay

Cellular radiation survival was determined following graded doses of 137-Cs radiation. Cells were irradiated in 25 cm^3^ flasks in exponential phase of growth. Colonies of > 50 cells were counted 10 to 14 days after plating. Clonogenic cell survival data were fit to the single hit, multi-target and the linear-quadratic models of radiation responses, as described previously [9, 10].

### *In vitro* irradiation experiments

DAOY cells were seeded in 6-well plates at a density of 200,000 cells per well 24 h prior to transfection, and maintained in 10% DMEM supplemented with 1% penicillin-streptomycin, and 1% L-glutamine for 24 h before transfection. Cells were transfected in serum-free DMEM, with 1% penicillin-streptomycin and 1% L-glutamine. At 24 h after transfection, cells were irradiated with a one-time radiation fraction of 10 Gy. Cells were incubated at 37°C for 24, 48 h and 72 h after irradiation. At the end of each incubation period, cells were collected for analysis of cell cycle distribution by flow cytometry. MTT analysis was done to determine the effect on cell growth 96 h post-transfection. Furthermore, the percentage of cells undergoing apoptosis was analyzed following EphB1 knockdown and ionizing radiation exposure 72 h post-transfection using an Annexin V detection kit (BD Pharmingen), according to the manufacturer's instructions. Analysis was done by Becton Dickinson FACS Calibur system.

### *In vivo* studies and radiosensitization experiments

An *EphB1^−/−^Smo* medulloblastoma mouse model was generated by breeding the ND2-SmoA1 mouse model (a generous gift from Dr. James Olson, Fred Hutchinson Cancer Research Center, Seattle, WA, USA) to the *EphB1^−/−^* mouse model [17]. All mice were kept, handled and euthanized in accordance with the ethics guidelines and conditions set and overseen by the Georgetown University Animal Care and Use Committee. The *EphB1^−/−^* mice were generated through the homozygous germline mutation of the *EphB1* allele [19]. The mice were genotyped as previously described [19]. The presence of the Smo A1 transgene cassette was verified by PCR as described previously [17] and by our group [16, 18]. The *EphB1^−/−^Smo* model therefore lacks EphB1 gene expression and expresses a constitutively activated form of Smoothened (SmoA1) gene in cerebellar granule precursor cells. The *EphB1*^+/+^*Smo* mice were used as controls.

Starting at postnatal day 23, tumor development in *EphB1^−/−^Smo* mice and their littermate *EphB1^+/+^Smo* controls (both age and gender-matched) was monitored by magnetic resonance imaging (MRI) [18]. Briefly, all MRI procedures were carried out in a 7T Bruker horizontal magnet run by the Paravision 5.1 software at the Georgetown-Lombardi Preclinical Imaging Research Laboratory. Quantitative tumor volumetric analyses were performed essentially as previously described [16, 18, 61]. The mice were anesthetized using 1.5% isoflurane and 30% nitrous oxide and positioned inside the magnet using a custom-designed animal holder with temperature and respiration control, which was further adapted to accept a Bruker 4 channel brain array coil. Anatomical MR imaging was performed with a two-dimensional T2-weighted RARE (rapid acquisition with relaxation enhancement) protocol with the following parameters: Matrix: 256 × 256, TR: 3500 ms, TE: 36 ms, RARE factor: 8, Averages: 4, FOV: 25 × 25 mm, and slice thickness: 0.5 mm.

### Allograft mouse model

Upon detection of a significant brain tumor mass, transgenic mice were euthanized by CO_2_ asphyxiation and cervical dislocation, in accordance with guidelines set by the Georgetown University Animal Care and Use Committee. Tumor masses of 100–200 mm^3^ were resected, placed in 4 ml of cell culture medium, and thoroughly minced. This cell suspension was mixed with Matrigel Matrix (BD Biosciences). For a tumor mass of approximately 200–300 mm^3^, 4 mL of cell culture medium was used. This suspension was combined with Matrigel at a ratio of 1:3 (v:v) respectively, and 200 μL of the suspension was injected subcutaneously into the mid-dorsal surface just rostral to the tail of nude mice.

Tumor masses were allowed to develop and volumetric tumor growth was measured using a caliper. Tumor volume was calculated using the following ellipsoid formula: (4/3)(π)(width axis radius × length axis radius × height axis radius). When tumor masses reached a size of approximately 50–100 mm^3^, the mice were randomized to control or treated groups and radiation was delivered (5 Gy/day × 4 doses). Tumor growth was monitored for 30 days after completion of radiation treatment. The numbers of tumor bearing mice in each group were: *EphB1^−/−^Smo* irradiated (*n* = 16), *EphB1^−/−^Smo* non-irradiated (*n* = 16), *EphB1^+/+^Smo* irradiated (*n* = 6), and *EphB1^+/+^Smo* non-irradiated (*n* = 6). Statistical significance of EphB1 deficiency on tumor growth curves was assessed using the GraphPad Prism 4.0 software (GraphPad Software, Inc.) by multi-linear regression analysis.

## SUPPLEMENTARY FIGURES


