# Supplementary material for: Knockdown of EphB1 receptor decreases medulloblastoma cell growth and migration and increases cellular radiosensitization
Source: Oncotarget. 2015 Mar 30;6(11):8929–46. doi: 10.18632/oncotarget.3369 (PMC4496193; doi:10.18632/oncotarget.3369)
Supplement: Supplementary file 1 [file oncotarget-06-8929-s001.pdf]

SUPPLEMENTARY FIGURES

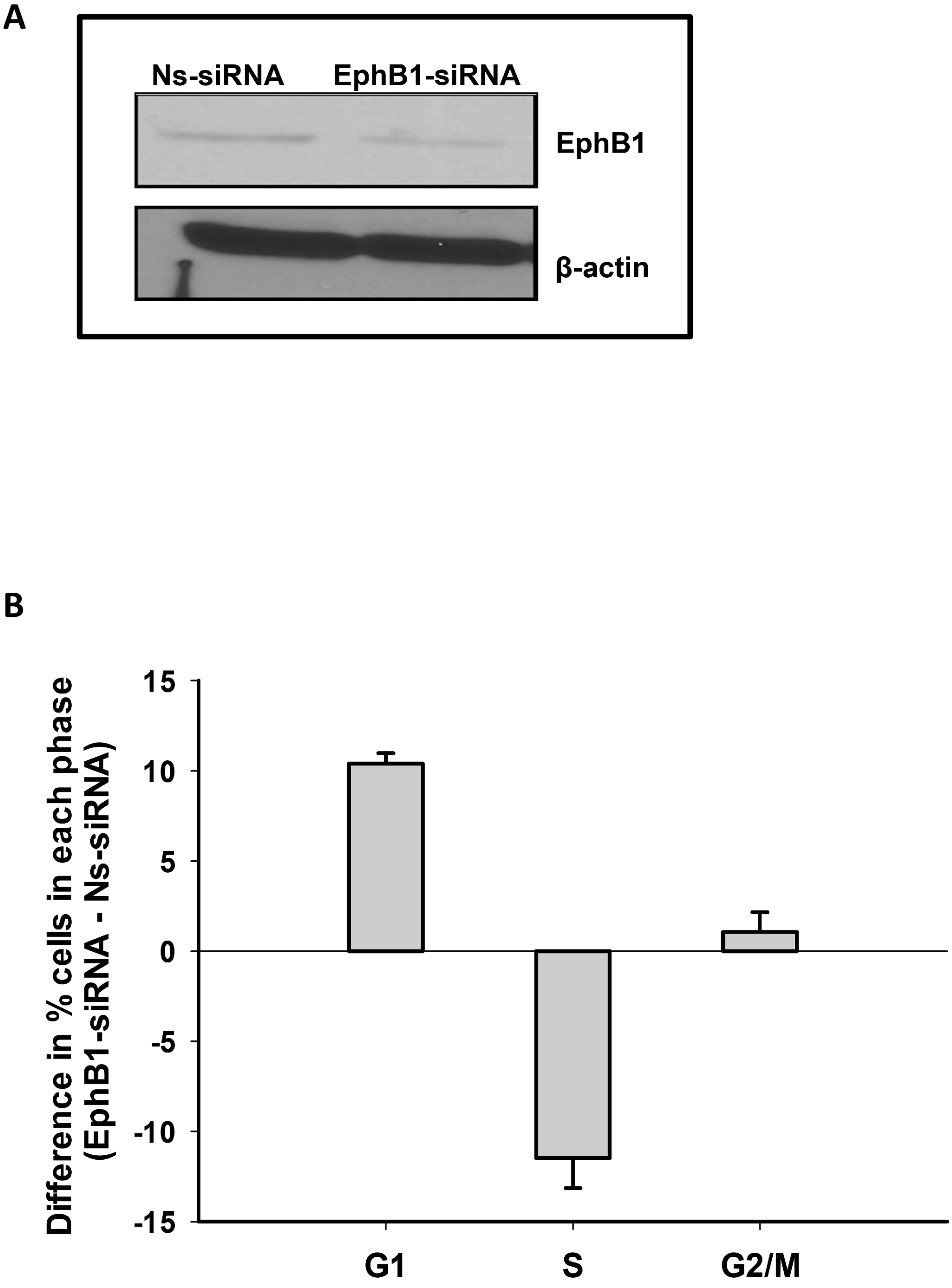

Supplementary Figure 1: EphB1 knockdown increases the percentage of cells in G1 phase in UW228 cells. (A) EphB1 expression is detected by western blotting in UW228 cells, which is decreased upon transfection with the EphB1-targeting siRNA vs. the control non-specific (Ns-siRNA). **(B)** Knockdown of EphB1 receptor increases the percentage of cells accumulated in G1 phase of cell cycle in UW228 cells. Data represent average values ± standard error from two independent experiments.

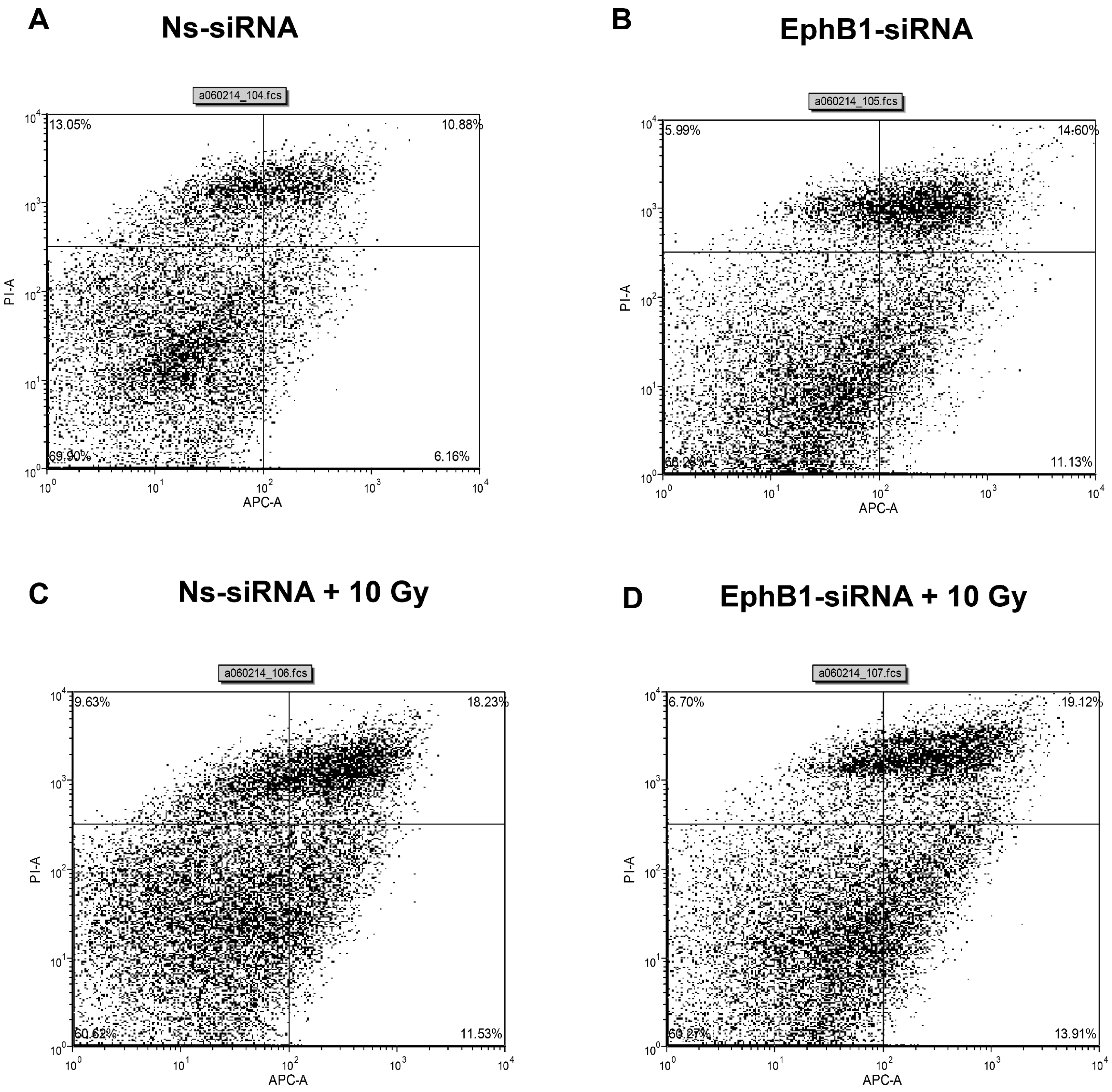

Supplementary Figure 2: Representative images from Annexin V/Propidium iodide (PI) staining show that EphB1 knockdown combined with ionizing radiation does not significantly enhance the number of Annexin V positive cells. DAOY cells were transfected with **(A)** non-specific siRNA (Ns-siRNA), **(B)** EphB1-siRNA, **(C)** Ns-siRNA with 10 Gy dose of ionizing radiation, and **(D)** EphB1-siRNA with 10 Gy dose of ionizing radiation. Cells were harvested 72 h post-transfection and analyzed by flow cytometry following Annexin V-PI staining.

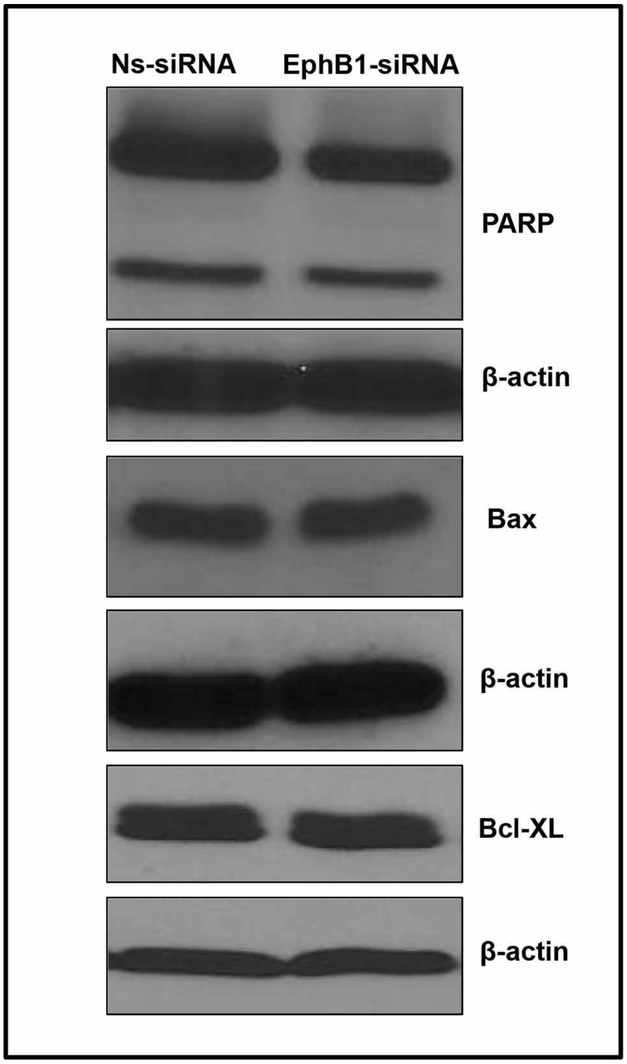

Supplementary Figure 3: EphB1 knockdown does not affect the expression of pro- and anti-apoptotic markers. DAOY cells were transiently transfected with non-specific siRNA (Ns-siRNA) or EphB1-targeting siRNA (EphB1-siRNA). Cell lysates were collected at 72 h post-transfection and probed against PARP, Bax, and Bcl-XL antibodies. No changes were observed in the expression of these protein markers following EphB1 knockdown.
